# Population genetics analysis of the Nujiang catfish *Creteuchiloglanis macropterus* through a genome-wide single nucleotide polymorphisms resource generated by RAD-seq

**DOI:** 10.1038/s41598-017-02853-3

**Published:** 2017-06-06

**Authors:** Jingliang Kang, Xiuhui Ma, Shunping He

**Affiliations:** 1 The Key Laboratory of Aquatic Biodiversity and Conservation of Chinese Academy of Sciences, Institute of Hydrobiology, Chinese Academy of Sciences, Wuhan, Hubei 430072 China; 2 University of Chinese Academy of Sciences, Beijing, 100049 China; 3 College of Animal Science, Guizhou University, Guizhou, 550025 China

## Abstract

Advances in genome scanning using high-throughput sequencing technologies has led to a revolution in studies of non-model organisms. The glyptosternoid fish *Creteuchiloglanis macropterus*, is widely distributed in the main stem and tributaries of the Nujiang River basin. Here, we analyzed IIB restriction-site-associated DNA (2b-RAD) sequences and mitochondrial DNA sequences, to assess the genomic signature of adaptation by detecting and estimating the degree of genetic differentiation among ten *Creteuchiloglanis macropterus* populations from the Nujiang River. The analyses revealed significant population differentiation among the up-tributaries, main stem, mid-tributary and low-tributary. Annotation of contigs containing outlier SNPs revealed that the candidate genes showed significant enrichment in several important biological process terms between up-tributaries and low-tributary, and exhibited prominent enrichment in the term macromolecular metabolic process between all tributaries and the main stem. Population dynamics analyses indicated that the Late Pleistocene glaciations strongly influenced the demographic history of *C. macropterus*. Our results provide strong evidence for the utility of RAD-seq in population genetics studies, and our generated SNP resource should provide a valuable tool for population genomics studies of *C. macropterus* in the future.

## Introduction

Population genetic studies are based on relatively wide distribution range. Such studies are helpful in detecting more significant differences to study their phylogeny and biogeography though they tend to focus on a low number of genetic markers^1–3^. However, when species have relatively narrow distributions, the detection of population differences is more difficult through classical methods, owing to insufficient data. In such case, classical methods often can-not meet the needs of researchers, who expect to detect intrinsic differences in population genetic studies. Recently, the rapid development of next-generation sequencing (NGS) has facilitated the identification of novel population genetic markers at an unprecedented scale, even in non-model organisms^4^. Thus, genome-wide patterns of diversity and differentiation can be explored without regard to whether the markers used are anonymous. Moreover, when thousands of loci across the genome are screened through high-throughput approaches, differentiation is generally detected even when only a small number of populations are considered^5–8^. These advances in genome scanning have allowed population genetics studies of non-model organisms^9^ and have improved understanding of evolution in ways that would not have been possible through classical methods^10^. Restriction-site-associated DNA sequencing (RAD-seq) has emerged as a powerful tool for genetic mapping and analyses of quantitative trait loci^4^, adaptation^11,12^ and phylogeography^13^. This method reduces the complexity of the genome, is less expensive and yields many more genetic markers than previous methods^14^.

To date, several population genetic studies of fish taxa have been conducted in the Nujiang River^3,15–17^. However, these studies have typically been limited to a few loci (rarely more than five loci) investigated using mitochondrial or nuclear markers. Most previous genome-wide studies have exclusively focused on endothermic terrestrial vertebrates^18–22^. Before the present study, the only similar studies conducted by Ma^23^ and Yang^24^, who performed transcriptome analyses detecting genomic signatures of adaptation to high altitude in glyptosternoid fish and schizothoracine fish. However, the identified adaptive signatures were based on the comparison with different species that inhabits a low altitude environment, rather than on within-species comparisons among different geographical populations.

*Creteuchiloglanis macropterus*, a glyptosternoid fish (Sisoridae, Siluriforms) that inhabits in cold waters, is mainly distributed upstream of the Nujiang River, which originates in the Tibetan Plateau and flows into the Southwest Mountainous Region of China^25,26^. This region functions as a transition zone where the altitude decreases to 1,000–2,000 m in Yunnan Province, China. Documenting the genetic mechanisms across environmental gradients in this area, might provide new insights into the evolutionary process of adaptation to the extreme conditions of the Tibetan Plateau. Glyptosternoid fish inhabit shallow, rocky rivers with moderate currents, and do not migrate long-distances. They tend to remain attatching to rocks in the rapidly flowing water unless they encounter sudden danger^23,27,28^. These special behaviors may lead to population differentiation. Therefore, we sought to conduct a population genomic analysis determine whether genomic signatures of adaptation exist among geographical populations of *C. macropterus*. Powerful evidence regarding the mechanism of adaptation to the Tibetan Plateau might be provided through the analysis of *C. macropterus* population genomics along gradient altitudes in the Nujiang River.

During the middle and late Pleistocene, glacial and interglacial periods caused repeated changes in the distributions of endemic fish on the Tibetan Plateau^29,30^. However, no study has investigated the population genetics and demographic history of *C. macropterus*. In our study, the population genetics of *C. macropterus* was assessed by using the IIB restriction-site-associated DNA (2b-RAD)^31^ tag technique. The objectives of this study were to (i) analyze the population genetic structure of *C. macropterus* across the Nujiang River; (ii) test whether adaptive signatures exist in the narrowly distributed populations, and, if so, identify the genomic regions of adaption by investigating genome-wide genetic variation and differentiation in ten populations; and (iii) explore the demographic history of *C. macropterus* and test whether the population dynamics has been influenced by climatic oscillations during the Quaternary.

## Results

### Mitochondrial DNA sequence data and phylogeographic structure

**Figure 1:**
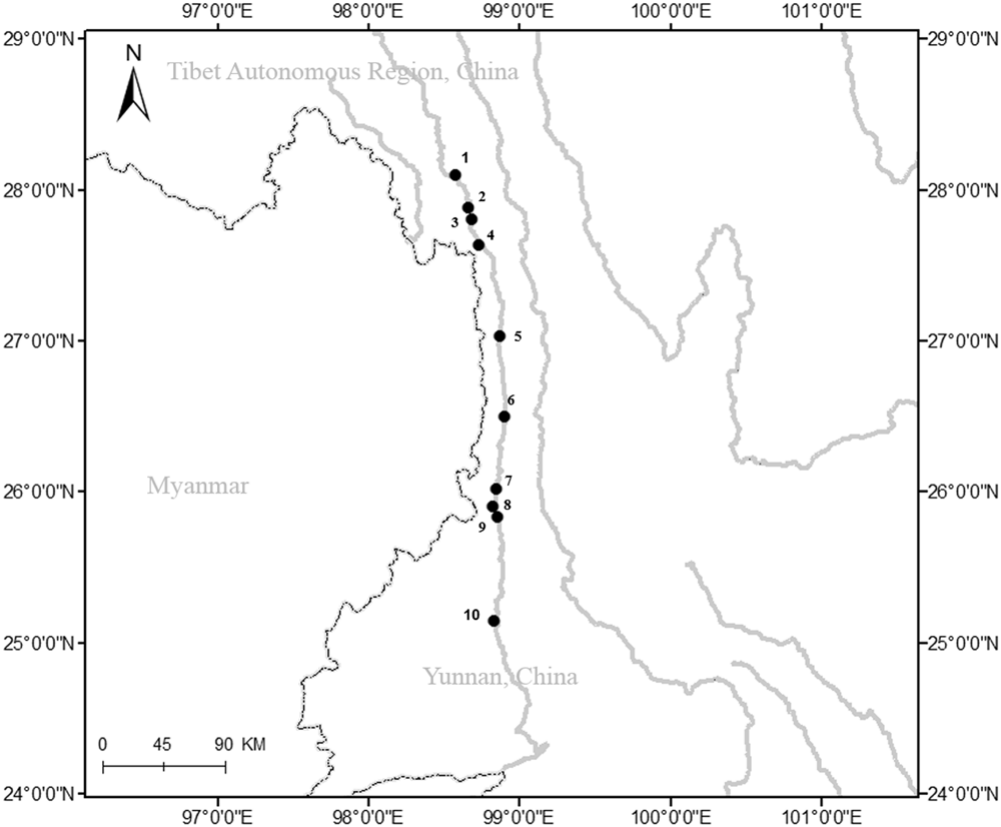
Map showing locations of the 10 sites where *C. macropterus* samples were collected. 1–10 are the codes of Qiunatong, Geza, Changwa, Xiyuegu, Lumadeng, Pihe, Delongba, Degeng, Liuku, and Mangliu, respectively. Of this, 1–4 are codes of up-tributaries, 5 and 10 are codes of mid-tributary and low-tributary, and 6–9 are codes of main stem. Map was created in the ArcGIS version 10.1 and modified in Microsoft Office.

Individual *C. macropterus* fish were sampled in the upper stream of the Nujiang River in Yunnan Province (Fig. 1, Table 1). Sequencing of the four mitochondrial DNA (mtDNA) segments with a joint alignment length of 2,983 bp in 102 *C. macropterus* individuals resulted in 49 haplotypes (Table S1). The GTR + I + G model with a proportion of invariant sites of 0.82 and a gamma shape parameter of 0.855 was selected for phylogenic analysis of the concatenated dataset.

**Table 1:** Sample collection data of Nujiang populations of *Creteuchiloglanis macropterus*.

Geographic region	code	coordinates	Altitude (km)	Collection date	N
Qiunatong	QNT	28°5′44″N, 98°34′43″E	2048	Oct/2015	22
Geza	GZ	27°52′24″N, 98°41′01″E	1515	Oct/2015	23
Changwa	CW	27°52′58″N, 98°39′58″E	1762	Oct/2015	18
Xiyuegu	XYG	27°38′11″N, 98°44′E	1706	Nov/2015	20
Lumadeng	LMD	27°1′57″N, 98°52′23″E	1334	Nov/2015	19
Pihe	PH	26°30′29″N, 98°54′11″E	1549	Mar/2013	23
Denglongba	DLB	26°01′18″N, 98°50′58″E	947	Mar/2013	22
Denggeng	DG	25°54′21″N, 98°49′37″E	942	Oct/2015	20
Liuku	LK	25°49′48″N, 98°51′37″E	815	Mar/2013	15
Mangliu	ML	25° 8′37″N, 98°50′9″E	874	Oct/2015	14
Total					196

The *N* is represented number of individuals sampled per locality, and the code is abbreviation of the geographic region.

The phylogenetic analyses based on Bayesian approaches (not considered sites with gaps) yielded two main groups (Fig. 1a): a tributary group, which contained haplotypes from the up-tributaries (UT), mid-tributary (MT) and low-tributary (LT); and a main stem (MS) group which contained 41 private haplotypes. In the tributary group, some haplotypes of mid-tributaries (LMD) formed a sister group with LT, and the other haplotypes of LMD clustered with UT. The median-joining networks (considered sites with gaps) (Fig. S1) also identified two main mtDNA haplotypes that corresponded to the tributaries and main stem, with no haplotypes shared between them. Among the tributary groups, one haplotype was shared among the three populations (CW, XYG and QNT) from the UT and one population (LMD) from MT in the tributary group, and one haplotype was shared by DLB and PH in the main stem. The main stem populations were characterized by high haplotype diversity (Table S1).

### RAD tag genotyping

A total of 697.2 million reads were derived from three pooled libraries that included 196 individuals. The mean number of reads per individuals was 3,557,185 (*SD* = 1921,313, range 247–13456,369). Of these reads, an average of 79% (*SD* = 12%, range 7–96%) carried the complete recognition site of BcgI enzyme. Raw sequencing data are available at BioProject SRP079503.

After exclusion of samples with few reads or few goal reads, the remaining 190 samples were used for the following analysis. The average number of stacks per individual was 47,076 (*SD* = 11,508, range 5,733–116,580), with the mean coverage per stacks ranging from 21 to 161X per individual. After merging of all the stacks from each individual, the final catalog contained 222,116 loci, of which 148,909 contained at least one SNP (92.3% biallelic, 6.4% triallelic, and 1.3% tetrallelic). Four SNP datasets (Table 2), with at least 80% of the individuals for all populations at >5X coverage per allele, were included in the following population analyses.

**Table 2:** 6 SNPs datasets constructed by the *populations* programs of STACKS.

SNPs Dataset	M	p	N_L_	Main application
1	10 (all ten populations)	10	1679	STRUCTURE, Bayescan
2	10 (all ten populations)	1	7125	BAYENV
3	6 (6 tributaries populations)	2	12125	Bayescan
4	3 (UT, LMD and ML)	3	8621	Sweep selective
5	2 (all tributaries (UT, MT and LT) and main stem (MS) as a group separately)	2	11363	Sweep selective

M and P are two main parameters of *populations*, M is the population map that determine which groupings to use for calculating summary statistics, p determines the minimum number of populations a locus must be present in to process a locus. N_L_ is the number of SNP loci in SNPs dataset, main application is the software that the dataset would be used.

### Population structure analysis

**Figure 2:**
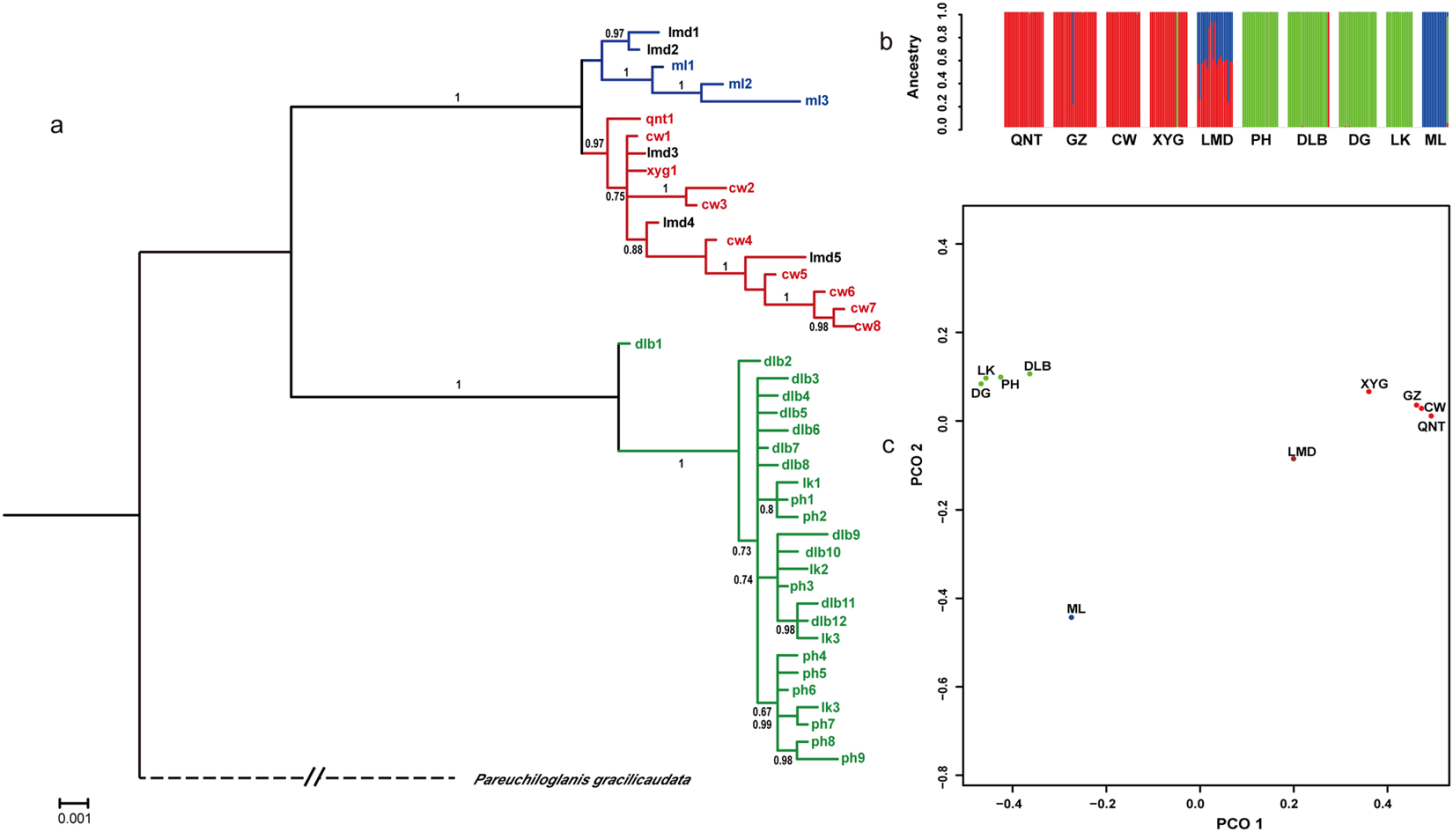
Genetic structure among the ten *C. macropterus* populations from the Nujiang river. (**a**) Bayesian tree constructed from concatenated mitochondrial haplotypes (2983 bp, GTR + I + G model, not considered sites with gaps). (**b**) Genetic clustering analysis for dataset 1 (1,679 SNPs), genetic clustering graph for the number of clusters (K = 3). Each color represents a different genetic cluster. Bar graphs present the average probability of membership (y-axis) of each individual. (**c**) Principal coordinates analysis (PCoA) plot of the overall average pairwise *F*_*ST*_ values of dataset 1.

A STRUCTURE analysis based on dataset1 (1,679 SNP loci) revealed Δ*K* = 3 (Fig. S2) to have the highest likelihood thus indicating three genetic clusters (Fig. 2b). Most individuals (Table. 1) in the up-tributaries (UT: QNT, GZ, CW and XYG) belonged to cluster 1 (in red), most specimens in the MS (LK, DLB, DG and LK) belonged to cluster 2 (in green), and most individuals in the low-tributary (LT: ML) belonged to cluster 3 (in blue). Clusters 1 and 3 both existed in the mid-tributary (MT: LMD) and exhibited almost equal average probabilities of membership. This arrangement was also indicated by the principal coordinate analysis (PCoA) plot (Fig. 2c) using the same dataset as that used in the STRUCTURE analysis, wherein the populations formed a distinct group along PCO 1. It was clear that four clusters (UT, MT, LT and MS) existed in the ten populations, and populations with close geographical distances always clustered together. Furthermore, LT and MT formed two distinct group, respectively. and MT was intermediate between LT and UT, thus indicating that MT might be a transition state of LT and UT. Together, the results indicate that the ten populations have a three-cluster genetic structure (comprising UT, MS and LT, and MT is an intermediate form between UT and LT).

The hierarchical AMOVA among the four clusters (UT, MT, MS and LT) resulted in Φ_ST_ = 0.82 (*P* < 0.001), indicating a very high level of genetic structure. A signal for isolation by distance was detected (*r* = 0.83, *P* < 0.001, Mantel’s test) among the ten populations. The results of the AMOVA and isolation by distance test revealed a pronounced genetic structure among the clusters, and the clusters followed a geographical pattern. In addition, the differentiation among the populations exhibited a closely correlation with geographical distance.

### Outlier analysis

**Figure 3:**
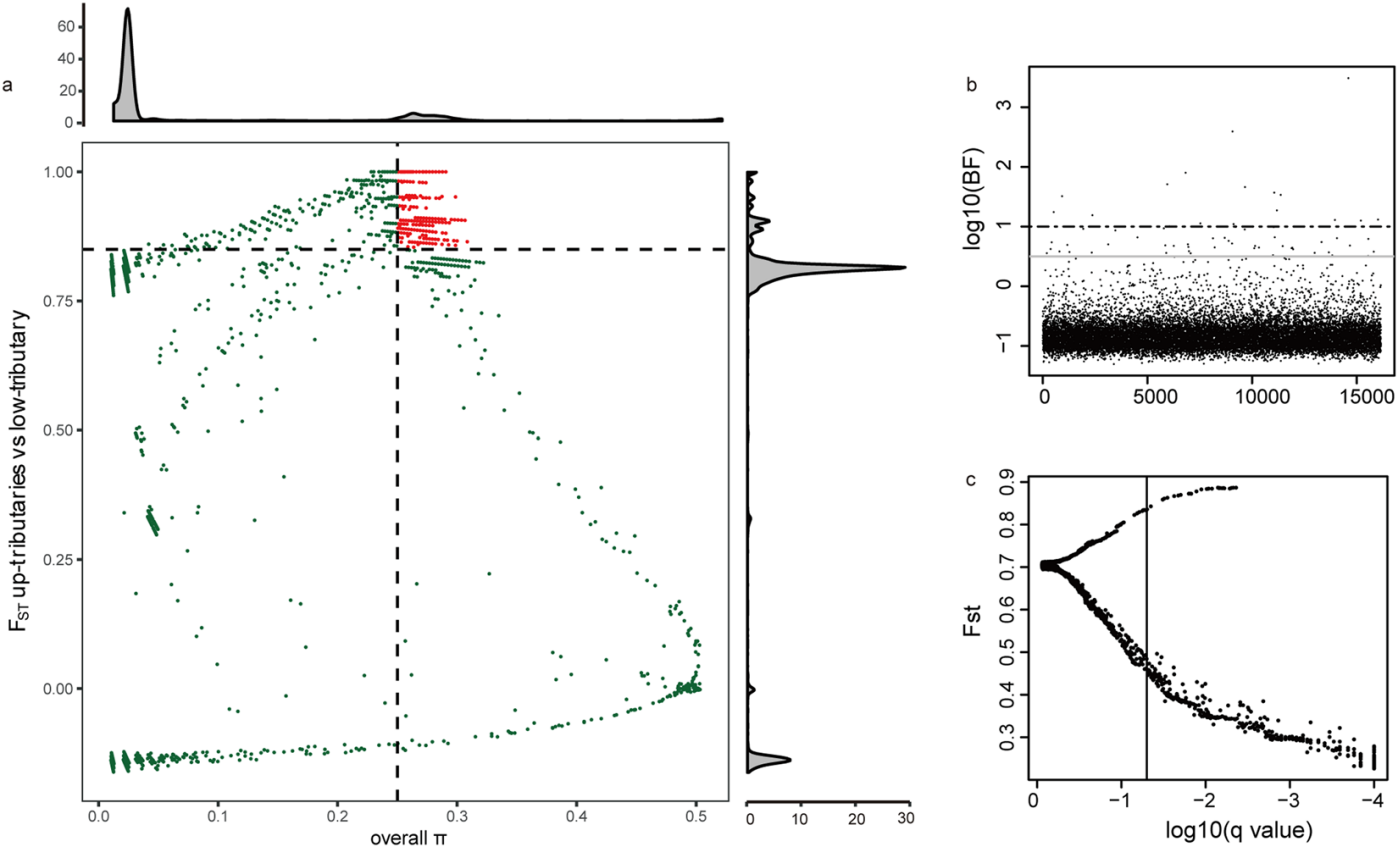
Outliers detection among tributaries. (**a**) Distribution of *F*_*ST*_ and overall *π* values of dataset 4 (8,621 SNPs dataset). Data points on the right of the vertical dashed line (corresponding to the 5% left tail of the empirical *π* distribution), and above the horizontal dashed line (5% right tail of the empirical *F*_*ST*_ distribution) were identified as selected regions for up tributaries and low tributaries (red points), and points represented by “+” are significant values. (**b**) Manhattan plot of genetic differentiation associated with altitude using dataset 2 (7,125 SNPs loci). Grey solid lines indicate lower thresholds of log_10_ (BF) = 0.5 and black dashed lines indicate higher thresholds of log_10_ (BF) = 1. (**c**) Global outlier detection among dataset3 (12,125 SNPs loci) in 6 *C. macropterus* populations (UT, MT and LT) from the Nujiang River. The vertical line represents a false discovery threshold of 0.01.

According to the genetic structure that was identified across all of the populations by using the 1,679 SNP loci in dataset1, the other three datasets were used to detecting selection footprints among the tributary populations. First, we ran Bayescan and identified 482 SNPs as outliers (39 directionally selected with relatively high *F*_*ST*_) among the 12,125 SNPs of dataset3, which were detected in the six tributary populations (Fig. 3c). Second, we pooled the samples of UT and LT together, and utilized the 8,621 SNP loci of dataset4 in UT and LT to identify the potentially adaptive loci in the tributaries. In total, we identified 881 loci with both a high *F*_*ST*_ and a high overall π, thus indicating that they might lie within a selective sweep region (Fig. 3a). Last, on the basis of the criterion of a log10 Bayes factor (BF) greater than 0.5, we found 53 SNPs (Fig. 2c) by BAYENV that were associated with altitude on the basis of the 7,125 SNPs of dataset2. After exclusion of loci that overlap with median *F*_*ST*_ in bayescan, a total of 957 loci were identified as outliers by using the aforementioned methods, and 10,746 loci were identified as neutral SNPs with Bayescan after removal of some outliers detected in the selective sweep and by BAYENV. To test for deviations from neutrality across UT and LT populations, Tajima’s D was estimated for this 957 loci by vcftools^32^, of which almost all were negative in UT (suggestive of positive or purifying selection) and almost all were positive in LT (suggesting balancing selection) (Fig. S3). And to ensure the tags with strong selective sweep, 553 tags with higher π in UT were remained in contrast with LT using VCFtools^32^. Preliminary comparisons with transcriptome indicated that 38 of the 553 RAD loci with SNP could be mapped to the transcriptome. Twenty-seven of these 38 contigs in the transcriptome resulted in significant hits (E-value < 1) in the nr database, and 22 had well-defined protein annotations (Supplementary dataset 1). And 399 loci were identified that might be in a selective sweep region between all tributaries and MS. However, there were no adaptive loci detected in Bayescan by using dataset1 (14 outliers (Fig. S4)).

**Figure 4:**
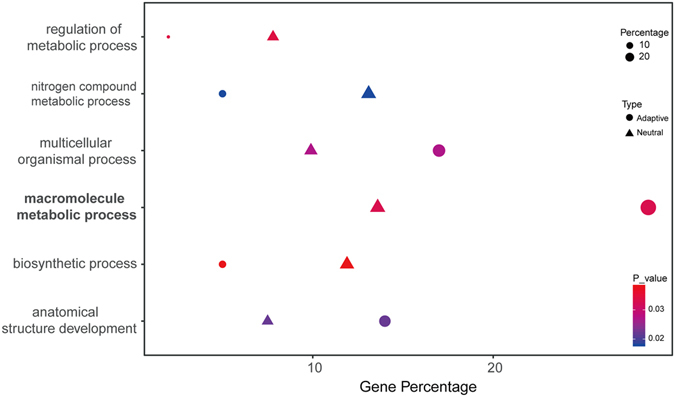
The significant enrichment GO terms for genes with adaptive SNPs and genes with neutral SNPs obtained with WEGO. The Bold GO term is detected between all tributaries and MS, the other GO terms are detected between up-tributaries and low-tributary.

Compared with genes with neutral SNPs, adaptive candidate genes showed a broad range of gene ontology (GO) annotations (Fig. S5 and Fig. S6). The enrichment analysis indicated that adaptive loci detected between UT and LT were significant enrichment in two functional categories (multicellular organismal process and anatomical structural development) (*P* < 0.05, Fig. 4). And annotation of these 399 adaptive loci detected between all tributaries and MS (22 had well-defined protein annotations) (Supplementary dataset 2) revealed significant enrichment in macromolecule metabolic progress as compared with neutral SNPs (*P* < 0.05, Fig. 4).

All of the genes involved in the above processes might have been important in promoting differentiation among the groups.

### Genes under selective sweep with high-altitude environments in UT

To identify genes that might be in correlation with the adaption to high altitude, we compared our candidate genes to an a priori list proposed^33^ which included 1,351 putative hypoxia-related genes. Finally, we identified four genes (SENP3, CTDSP1, BMPR2, MAP1LC3B) that were consistent with the list, and five genes (USP20, GIMAP8, PARP14, DNAJB5, SOCS1) that might be homologous or analogues in contrast with the genes in the list (Table 3). These nine genes were found to most likely be involved in the response to hypoxia and oxygen binding. For example, the stabilization and redistribution of SENP3 (sentrin-specific protease 3-like) correlate with an increase in the transcriptional activity of the hypoxia-inducing factor1 (HIF-1) under mild oxidative stress^34^, which are consistent with the medium elevation of UT. Reduced BMPR2 (bone morphogenetic receptor type-2) expression occurs in chronic hypoxic rat models of pulmonary arterial hypertension (PAH)^35^. DNAJB5 (dnaJ homolog subfamily B member 5-like) belongs to the DNAJ family, which is a heat shock protein 40 (Hsp40) family protein, their expression is significantly up-regulated under stresses of hypoxia^36,37^. These positive selection genes indicate that populations in UT are more adaptive with hypoxia than populations in LT.

**Table 3:** Positively selected genes involved in the hypoxia response in *C. macropterus* (UT populations).

Gene name	Description
**SENP3**	sentrin-specific protease 3-like
**CTDSP1**	carboxy-terminal domain RNA polymerase II polypeptide A small phosphatase 1-like
**BMPR2**	bone morphogenetic receptor type-2
**MAP1LC3B**	microtubule-associated 1A-like
USP49	ubiquitin carboxyl-terminal hydrolase 49
GIMAP8	GTPase IMAP family member 8-like
PARP14	poly [ADP-ribose] polymerase 14-like isoform X1
DNAJB5	dnaJ homolog subfamily B member 5-like
SOCS1	suppressor of cytokine signaling 1-like 489

Gene names in bold are completely consistent with the 1,351 putative hypoxia-related genes, the others are homologous or analogues.

### Population demographic history

**Figure 5:**
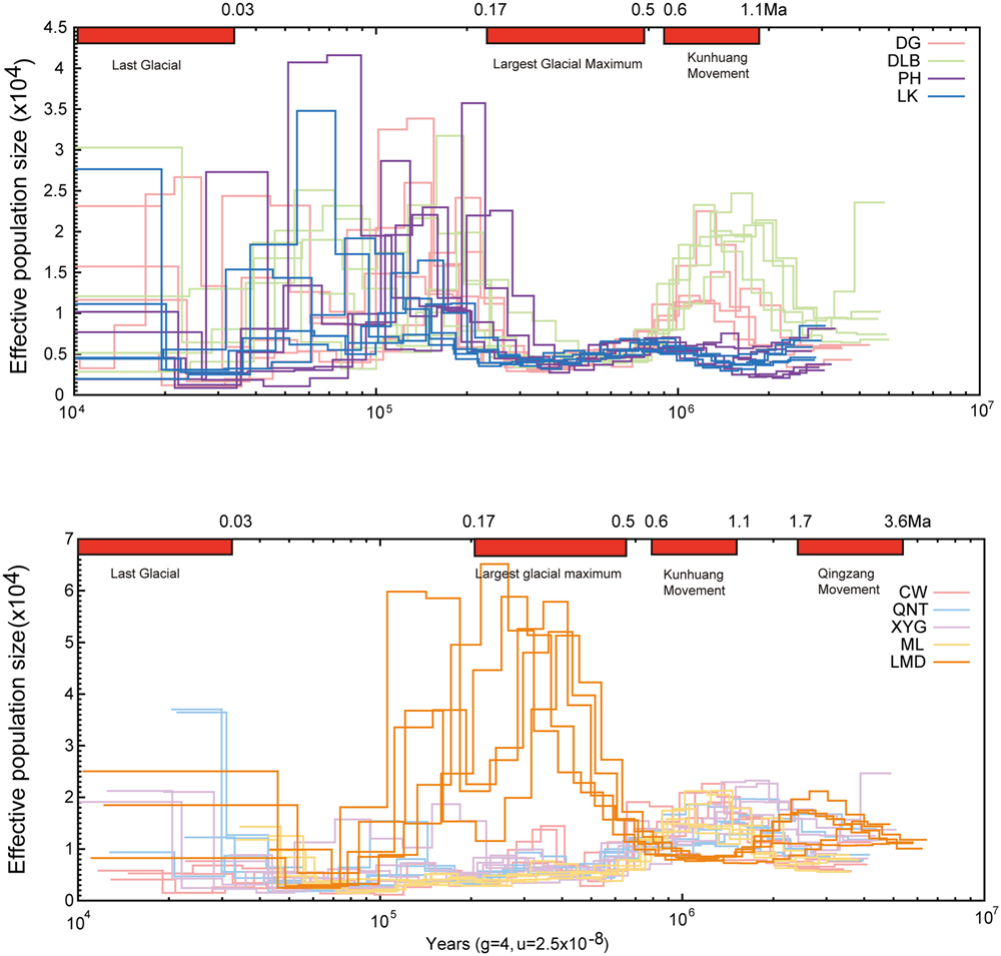
Inference of ten *C. macropterus* populations demographic history. each thin line represents one individual, each population is represented by five individuals. The above is the effective population size of populations in MS, and the below is the effective population size of populations in all tributaries.

We estimated changes in effective population size by using a Pairwise Sequentially Markovian Coalescen (PSMC) method^38^ to explore the demographic history of *C. macropterus* populations from the main stem and tributaries, respectively. It seems that the population demography has a correlation with the uplifting of the Tibetan Plateau^39,40^ (Fig. 5, Fig. S6). The up and low tributaries populations underwent approximately decline from 1.2 Ma (Fig. 5) during Kunhuang movement (1.1–0.6 Ma). However, as a tributary population with middle altitude, the decreasing of effective population size in LMD occurred earlier (about 1.8 Ma) during Qingzang movement (3.6–1.7 Ma), and the results showed effective population size of LMD reduced to a low level during Kunhuang movement. Interesting, LMD population has an expansion where the other tributaries had not exhibited. Among main stream populations, two populations (DG, DLB) of MS showed a constriction during 1.2–0.47 Ma, and the other two (PH, LK) showed a constriction during 0.69 to 0.24 Ma. Besides, the results also showed that the effective population size of the *C. macropterus* populations fluctuated during cyclical climate changes in the Tibetan Plateau (Fig. S7). All populations (excluding LMD) were keeping a low effective population size during the largest glacial maximum (LGM), and LMD became decreased at the LGM, which occurred from the middle Pleistocene (0.5 Ma) to 0.17 Ma^41–43^. The last glacial (LG), which started at approximately 0.075 and continued until 0.01 Ma^44,45^, seemed have no evident effect on *C. macropterus* populations.

Bayesian skyline plot (BSP) analyses also indicated that all of the populations experienced a decline during the glacial period (Fig. S8). Populations from MS showed a decline from approximately 0.07 to 1.1 Ma, and the same scenario occurred within 0.07–0.71 Ma in populations from tributaries.

## Discussion

In the present study, we identified a large number of SNPs for *C. macropterus* by using the RAD-seq approach. The key finding of this study was the high degree of genetic differentiation due to divergent selection among *C. macropterus* populations in different regions of the Nujiang River. This finding suggest that adaptive differentiations were apparent in local populations. Annotation of the outliers confirmed the existence of potential adaptive genomic regions. Furthermore, demographic analyses of the ten populations confirmed that the *C. macropterus* populations were influenced by Quaternary climatic oscillations. We discuss these findings and compare them with those of earlier studies on the genetic mechanisms of adaptation in the environment of the Tibetan Plateau below.

### Genome-wide differentiation among *Creteuchiloglanis macropterus* populations

Our RAD-seq analysis revealed that the overall degree of genetic differentiation among the *C. macropterus* populations was fairly high (average pairwise *F*_*ST*_ = 0.578 across all populations) on a genome-wide scale (Table 2), and the populations fit the model of isolation by distance. However, genetic differentiation among the UT or MS populations was consistently low (average pairwise *F*_*ST*_ = 0.0318 across UT populations; average pairwise *F*_*ST*_ = 0.0025 across MS populations), a result consistent with the mtDNA results (Table S2).These findings were similar to the previous estimates of genetic differentiation in *Glyptothorax zainaensis*^46^ on the basis of Cytb. Given that the UT populations are separated by mountain, and that the MS populations could disperse more readily, it is reasonable that the observed pairwise genetic differentiation among the UT populations was higher than that among the MS populations. Low differentiation among these populations might have resulted from the homogeneous environment in proximity to the sampling localities. Genome-wide differentiation among the populations provided robust evidence that *C. macropterus* can easily form distinctive geographical populations, and spatial distance promoted genetic differentiation among these populations. The statistically significant and high *F*_*ST*_ value showed that differentiation occurred not only among the tributary populations, but also between all tributaries and MS populations.

Local adaptation analyses of *Creteuchiloglanis macropterus* using different SNP datasets. Despite the low degree of genome-wide genetic differentiation between the UT and MS clusters, we identified a large number of loci with very high differentiation by scanning the *F*_*ST*_ values of the loci in the pairwise populations. CW and GZ, nearby populations in UT, exhibited a low *F*_*ST*_ value (0.043) (Table 4). However, we identified 22 loci with high *F*_*ST*_ values (>0.15) in dataset 2. In addition, despite extremely low *F*_*ST*_ values (−0.001) (Table 4) between LK and DLB, 21 loci were detected with high *F*_*ST*_ values (>0.15). These findings demonstrate the power and potential of RAD tags^10^ to detect subtle differentiations in population genetics^15,16^.

**Table 4:** Slatkin pairwise *F*_*ST*_ values among all sampling localities of *C. macropterus* populations.

code	1	2	3	4	5	6	7	8	9	10
1.qnt	—	26.48	29.78	53.65	121.61	181.63	231.69	245.59	254.20	328.35
2.gz	**0.043**	—	3.74	27.47	98.11	155.70	209.49	221.68	232.76	311.83
3.cw	**0.022**	0.043	—	23.15	93.31	151.12	199.39	212.55	225.23	304.22
4.xyg	**0.041**	0.020	0.022	—	68.55	128.35	181.59	192.31	204.79	283.62
5.lmd	**0.449**	**0.383**	**0.280**	**0.385**	—	59.31	114.67	127.32	135.49	211.84
6.ph	**0.906**	**0.867**	**0.899**	**0.849**	**0.671**	—	56.76	66.96	76.06	151.67
7.dlb	**0.857**	**0.823**	**0.848**	**0.800**	**0.643**	−0.001	—	12.39	22.82	98.96
8.dg	**0.915**	**0.872**	**0.909**	**0.854**	**0.664**	**0.011**	−0.011	—	10.42	86.28
9.lk	**0.918**	**0.873**	**0.911**	**0.854**	**0.658**	**0.005**	0.009	0.002	—	72.29
10.ml	**0.941**	**0.903**	**0.941**	**0.897**	**0.516**	**0.886**	**0.847**	**0.882**	**0.886**	—

Bold numbers are significant values under α = 0.05. Above diagonal are geographic distances between populations (km).

With respect to the differences among the three tributaries groups (UT, MT and LT), we found significant difference in two biological processes between the outliers and neutral SNP loci (Fig. 4). These processes are associated with metabolism which might be very important at the high altitude. The percentage of genes in the tributaries that participated in the term macromolecule metabolism process was clearly higher than that in MS (Fig. 4). The differentiations among tributary groups or between the tributaries and MS both were related to the metabolic process. Compared with LT populations, UT populations at higher altitudes need more metabolic activity to cope with the relatively poor environment^23,47^. The populations from the tributary were found in high mountains areas and are subjected to a colder water environment than are the MS populations, and they therefore might require more energy. And the difference elevation accelerated the genetic structure differentiation between UT and LT, the positive selected genes of populations in UT were associated with hypoxia in order to adapt the higher elevation environment.

The finding that only small percentages of the outliers (10.45% and 8.47%) were mapped to the assembled transcriptome, may be due to the few BcgI enzyme recognition sites that occurred in the protein-coding regions of the DNA in *C. macropterus*. In the condition of lacking genome, lots number of loci would be filtered even they locate in non-coding region of positive selected genes. And in consideration of the low mapping ratio, we combined empirical method, Bayescan and Bayenv to detect outlier loci as much as possible. Therefore, the number of outliers were enormous which might be redundant. Our results suggest that the genetic structure of these populations is also driven by variation in environment conditions rather than by geographic alone, however, we only detected outlier loci that appeared to be driven by selection associated with spatial heterogeneity in altitude. The associations with environmental parameters and footprints of selection may be derived from some unmeasured factor (e.g., ecological, physiochemical)^48^, which might omit some outlier loci. It might be improved by measuring more environmental factors (e.g., temperature, characteristic of water) of sample localities. *C. macropterus* is mainly distributed upstream (UT, 83 individuals) and scarce in low-elevation location (LT, only 14 individuals), and we sampled the outlier loci with high value of overall π in the empirical method, these π value of loci might only exist in UT but not LT which would lead to omit some loci, it would be improved if we could increase the number of individuals in LT. In addition, when it exists the recent admixture between populations, some shared loci that should have high *F*_*ST*_ would be filtered because of their low values of *F*_*ST*_.

### Demography of *Creteuchiloglanis macropterus*

The demography of species around the Tibetan Plateau has drawn mach attention because of the region’s unique paleogeographical history and paleoclimatic oscillations. The Tibetan Plateau uplifted approximately 3000 m and experienced at least four major glaciations during the Pleistocene^30,42^. This uplift and the associated climate changes are considered to be the most important factors influencing the current spatial distributions, genetic diversities and population structures of local species^49–51^. In our study, we found that the effective population sizes of all *C. macropterus* populations might underwent decreases caused by uplift of The Tibetan Plateau. The patterns of coincidence population declines in these population might be a response of a common background following the intense uplift of the Plateau that may have been unfavorable to them^52^. It is strange to detected that all populations were maintained at a fairly low level while LMD has a relatively higher population size, which might be involved with the more feasible environment constructed by uplift of the Tibetan Plateau in LMD. Moreover, in contrast to the increase of MS effective population size after the period of the LGM, all tributaries were retaining a low level of effective population size after Kunhuang movement. In consideration of individuals of ML were sample in high mountain, the reason might lie in that *C. macropterus* migrated to low-altitude with warmer environment. Most temperate species appear to have dispersed to lower latitudes in response to Pleistocene climatic oscillations^53,54^.

In summary, all populations showed low effective population size or a decreasing tendency in the LGM, which have not detected in the LG, it indicated that *C. macropterus* in the Nujinag river were mainly effected by the LGM rather than the LG. The reason might be the habitat of *C. macropterus* might not have been covered by ice during the LG. *C. macropterus* in our study dwell at 815–2048 m, while the snowline was 1800–3200 m^15,55,56^. However, the BSP analysis of the mtDNA dataset showed that all of populations along the Nujiang basin were suppressed during the time that spanned the LGM and LG. The reason for these results might be that the use of a limited number of loci decreased the resolution of the results^57^.

## Conclusions

The present study combined analyses of mtDNA and RAD-seq datasets, and provides the first exhaustive survey of genome-wide genetic variability and differentiation in Chinese glyptosternoid (*Creteuchiloglanis macropterus*) populations. Genomic heterogeneity at the level of genetic differentiation appears to be associated with adaptation to the high-elevation environment, we first detected the hypoxia adaption of fish in population genomic scale. This study also demonstrates that RAD-seq is a powerful tool for obtaining more genome information, it can provide much more genome information than is obtainable through classical methods. The SNP resources generated in this study might be valuable for future population genetics and genomics studies of *C. macropterus*. In addition, this study provides the first evidence that the population genetics and dynamics of *C. macropterus* were profoundly influenced by Quaternary climatic oscillations.

## Methods

All the methods were carried out in accordance with approved guidelines. All experimental protocols involving animals in this study were approved by the Ethics Committee of the Institute of Hydrobiology, Chinese Academy of Sciences.

### Sampling and DNA extraction

Individual *C. macropterus* fish were sampled in the upper stream of the Nujiang River in Yunnan Province (Fig. 1, Table 1), and the sample identification was performed as described by Zhou and Chen^25,26^. The Pihe (PH), Denglongba (DLB), Denggeng (DG), and Liuku (LK) populations were sampled in the main stem of the Nujiang River. The Qiunatong (QNT), Geza (GZ), Changwa (CW), Xiyuegu (XYG), Lumadeng (LMD), and Mangliu (ML) populations were sampled in the tributaries. Caudal fin or muscle tissue from each fish was preserved in 95% ethanol for DNA extraction. Then, the samples were maintained in 10% methanol for later identification and were deposited in the Freshwater Fish Museum of the Institute of Hydrobiology at the Chinese Academy of Sciences. Total genomic DNA was extracted using a standard procedure (E-Z-N-A Tissue DNA kit; OMEGA Bio-tek).

### Mitochondrial DNA sequencing

Four regions of mitochondrial DNA sequence (cytochromec oxidase submit I (COI), cytochrome b (Cytb), control region (CR) and NADH dehydrogenase submit 5 (ND5)) were used as a mitochondrial concatenated data set to construct a phylogeny for verifying the genetic structure that was detected by RAD-seq. Polymerase chain reaction (PCR) amplifications were carried out using the following primers: FishF1 and FishR1^58^ for COI, L14724 and H15915^59^ for Cytb, GEDL200 and GEDH860^60^ for CR, and ND5-F(GCATCCTGATACATACACTCCGA) and ND5-R (TGTTTGGAGGCTGTATTGGCT) for ND5. Each PCR tube contained approximately 100 ng of template DNA, 1 µL of each primer (10 pmol/µL), 3 µL of 10 × reaction buffer, 1.5 µL of dNTPs (2.5 mmol/L each), and 2.0 U of Taq DNA polymerase in a total volume of 30 µL. The PCR conditions were as follows: initial denaturation at 94 °C for 3 min, followed by 30 cycles of at 94 °C for 1 min and annealing at 58–64 °C for 1 min, followed by a final extension at 72 °C for 5 min. Finally, we obtained COI (accession number KY232646–KY232771), Cytb (accession number KY232772–KY232889), ND5 (accession number KY232890–KY233000) and CR (accession number KY233001–KY23319) sequences with a joint alignment length of 2,983 bp (COI: 567, Cytb: 1100, ND5: 837 and CR: 479) in 102 *C. macropterus* individuals.

### 2b-RAD library preparation and Illumina sequencing

Three 2b-RAD sequencing libraries were prepared at the Institute of Hydrobiology at the Chinese Academy of Sciences (Wuhan, China), as described in Wang^61^. The type IIB restriction enzyme chosen for the experiment was BcgI (New England BioLabs), which excises a 36-bp fragment around the recognition site, cleaves genomic DNA upstream and downstream of its target site, and generates tags of uniform length that are ideally suited for sequencing on existing NGS platforms. Digestion reactions were performed in a total volume of 6 µL, using 4 µL of intact, high-quality genomic DNA sample, each containing a total of 0.25 to 1 µg of DNA in 4 µL, 1 unit of BcgI, 0.6 µL of 10 × NEBuffer 3 and 0.4 µL of 150 µM SAM. The reaction was inactivated at 65 °C for 20 min, and the reaction tubes were then incubated at 37 °C for 2 h. Then, 1 µL of the digested DNA was loaded to verify the effectiveness of the digest. Subsequently, 20 µL of ligation master mix containing 0.5 µL 10 mM ATP, 800 U of T4 DNA ligase (New England BioLabs), 2 µL of 10 × T4 ligase buffer, 1 µL of each adaptor (5 µM) and 14.5 µL nuclease-free water (NFW) was added to the remaining 5 µL of digested DNA. Each reaction tube was incubated at 16 °C for at least 2 h and then stored at 4 °C overnight.

The ligation products (16 µL) were amplified in 80 µL PCRs, each containing 1.6 µL of each primer (IC1-P5, IC1-P7), 10 µL of 10 × dNTP, 1.2 mM Mpx primer, 0.8 µL (1.6 U) of Phusion high-fidelity DNA polymerase (New England BioLabs), 16 µL of 5 × HF buffer and 20.8 µL of NFW. The reaction protocol consisted of 17 cycles at 98 °C for 20 s, 65 °C for 2 min and 72 °C for 30 s, followed by a final extension of 72 °C for 10 min. Each individual reaction had a unique barcode to distinguish itself. The final genomic libraries were selected for 170 bp by gel extraction (Qiagen). A total of three multiplexed libraries, including 196 samples, were sequenced with 37, 86 and 72 samples in the three libraries. The resulting fragments were sequenced on an Illumina HiSeq 2500.

### RAD tag genotyping

Before the *de novo* analysis, custom Perl scripts were used to obtain the reads with complete BcgI recognition sequences (https://github.com/z0on/2bRAD_denovo). First, we discarded individuals with obviously fewer reads than the others. Then, the reads were trimmed to a length of 32 bp, and quality filtering (Phred score more than 20 for at least 93% of the bases in the read) was performed using the FASTX TOOLKIT. The remaining reads were then analyzed in the software pipeline STACKS v.1.23^62^.

All of the reads were pooled and used for *de novo* assembly in *ustacks* (STACKS pipeline)^62^. We set a minimum stack size of five reads (*m* = 5) and excluded stacks with coverage lower than this threshold. Invariant stacks were then compiled into sample-specific loci if they differed by fewer than two nucleotides (*M* = 2). Then, sample-specific loci were assembled into homologous loci if they differed by fewer than three nucleotides (*n* = 3) between samples, by using *cstacks*^63^. Finally, the *populations* program^64^ was used to obtain the loci that satisfied the following conditions: (i) the loci were present in at least 80% of the individuals from each population, and (ii) at least five RAD tags were present per allele at each locus. To limit false single-nucleotide polymorphism (SNP) identification, we removed SNPs with a global minor allele frequency <0.05^65^. Furthermore, potential homologs were excluded by removal of markers that exhibited heterozygosity values >0.5^66^. To avoid linkage bias for the SNP calling, only the first SNP per locus was included in the final analysis^67–71^. To maximize the detection of adaptive loci, a series of SNP datasets (Table 2) were constructed in the *populations* program in STACKS on the basis of genetic structure, by specifying parameters in the *populations* program (-p: change the number of populations that loci should be present; -M: population map, determine which groupings to use). In general, genomic regions exhibiting high *F*_*ST*_ values may represent signatures of local adaptation or islands of differentiation resistant to gene flow^72,73^. Loci with high *F*_*ST*_ and high diversity values were selected by two-model-contrasting analysis. Furthermore, potential adaptive loci were detected by using additional dataset, and these loci were eventually combined and contrasted with the neutral loci detected in Bayescan which was used to remove the potential adaptive loci.

### Phylogeographic and population structure analysis

COI, Cytb, CR and ND5 partial sequences from the *Pseudexostoma brachysoma* mitochondrial genome were used as out-group sequences because of the close phylogenetic relationship of this species with *C. macropterus*^47^. The homogeneity test in PAUP* 4.0b10^74^ was conducted on the four concatenated gene haplotype sequences, and no significant heterogeneity was detected (*P* = 0.21, 1000 replicates). Models of nucleotide substitution were tested using jModelTest 2.0^75,76^, and a corrected Akaike information criterion (AICc) was used to determine the best-fit model. Bayesian trees were constructed with MRBAYES v.3.1.2^77^ to generate Bayesian posterior probabilities for phylogenetic inferences. The closest general model was used when specific models selected by jModeltest were not implemented in MrBayes. Two independent Markov chain Monte Carlo (MCMC) runs, each with six chains for 5 million generations, sampled every 1000 steps, were performed. We confirmed convergence to a stationary distribution by determining whether the deviation of split frequencies was below 0.01 between the two independent runs, and we discarded the first 1,250 trees as burn-in.

We used STRUCTURE v.2.3.3^78^ to estimate the population-genetic clusters by using dataset 1 with the hash term that all of the the SNP loci should be present in all of the ten populations. For this analysis, we assumed an admixture model and correlated allele frequencies with no prior information. We used a burn-in of 10,000, followed by another 10,000 MCMC steps. Then, we estimated the most likely number of clusters by estimating the maximum likelihood for *K* = 1–10, and ran each cluster option five times. The optimal *K* value was selected on the basis of the *L(K)* values; the individual assignment patterns using STRUCTURE HARVESTER Ver. 0.6.93^79^, which assesses the likelihood value at each K and selects the optimal value by using the ad hoc statistic Δ*K*; and the rate of change in the log probability of data between successive *K* values^80^. To visualize the multi-locus patterns of population differentiation, a PCO plot was generated using the R package labdsv^81^, on the basis of average *F*_*ST*_ values.

Hierarchical AMOVA was conducted among clusters of sampling localities identified by STRUCTURE and PCO using Arlequin v.3.5^82^, and NJ. Slatkin^83^ pairwise *F*_*ST*_ values were also estimated in Arlequin. The patterns of population differentiation were compared using a Mantel’ test^84^. Tests for isolation by distance were conducted using a Mantel’ test with linearized *F*_*ST*_ values [*F*_*ST*_/(1 − *F*_*ST*_)] and geographic distances separating sampling locations. Significance was tested after 1,000 permutations. Network 5.0^85^ was used to reconstruct the phylogenetic relationships among the haplotypes with a median-joining network method.

File conversion of the STACKS output files to the desired formats was carried out with PGDSPIDER Ver.2.0.8.0^86^.

### Outlier analysis

To identify genomic regions that showing marked genetic differentiation among the tributaries, we constructed dataset 4, in which the tributaries were divided into three groups (UT, MT and LT). The allele frequencies of the variable sites were used to identify regions that were potentially affected by long-term selection, by using two complementary approaches^87^. We calculated population fixation statistics (*F*_*ST*_) and the nucleotide diversity (π) between UT and LT for each SNP loci in dataset 4. Similarly to the empirical outlier detection approach^12,88,89^, the putative selection targets were extracted on the basis of their representation in the top 5% of the odds ratio for both *F*_*ST*_ and π. In addition, Bayescan 2.1^90^ was used to estimate the posterior probability that a given locus was affected by selection using dataset 3. Bayescan was run under the default settings, and the false discovery rate (FDR) was set at 0.05. In addition, we used the same method with dataset 1 and dataset 5 to identify genomic regions that showing marked genetic differentiation between the tributaries and MS.

To investigate whether allele frequencies at any individual SNP locus were correlated with altitude and to prioritize the detect on of such SNP loci, a Bayesian approach was implemented in BAYENV^91^, to filter the SNP dataset 2 identified by STACKS, wherein all of the populations were taken into consideration; and any loci present in at least one population included in this dataset. Thus, we were able to maximize the detection of the SNP loci that were correlated with altitude. The altitude data were standardized by subtracting the mean and dividing by the standard deviation of the parameters across all of the sites in dataset 4.

We used the empirical approach (dataset 5) and Bayescan (dataset 1) respectively to identify the differentiated genomic regions between tributaries and main stem populations. And to identify the putative functions of the outlier loci, a *C. macropterus* reference transcriptome (unpublished) was used to annotate the protein-coding outlier loci. For each locus, a strict majority consensus sequence was generated and aligned to the *de novo* transcriptome using Bowtie 1.1.2^92^ allowing up to one mismatch. Putative gene identities were determined by using homology searches with BLASTX against the nr database from the NCBI website; in the case of multiple hits, the best match was chosen. Then, functional annotation of these genes performed using the BLAST2GO suite (http://www.blast2go.com/b2ghome)^93^. Only those ontologies with an E-value < 1E-6, an annotation cut-off >55 and a GO weight >5 were considered for annotation.

### Inference of demographic history

Demographic history was reconstructed on the basis of a Hidden Markov Model (HMM) approach using PSMC^38^. Using the consensus loci created by the *cstacks* program, we concatenated the tags of each locus as the reference genome. Briefly, the RAD tags of each sample were aligned to the reference genome using BWA^94^ (version 0.7.3a-r367) with the default settings. The consensus sequences were called using SAMtools^95,96^ (version: 1.3.1). The ‘fq2psmcfa’ tool was used to create the input file for the PSMC modeling, with the option –q20. The resulting files were used as the input for the PSMC estimates using ‘psmc’ with the options −N25 −t15 −r5 −p’4 + 25*2 + 4 + 6’. The reconstructed histories of each population were plotted using ‘psmc_plot.pl’ with generation time (-g 4) and mutation rate (-u 2.5e-08). The mutation rate was selected according to the rates described in previous studies in medaka^97^ and channel catfish^98^. Historical population dynamics were also estimated from the mtDNA dataset by using coalescent-based Bayesian skyline plots (BSPs)^99^. BSPs were generated using BEAST 2.3.2^100^ to describe the demographic history by assessing the time variation of effective population size, and the results of the BSPs were visualized in Tracer 1.5.

## Electronic supplementary material

supplementary information

Dataset 1

Dataset 2
