# Author Correction: Population genetics analysis of the Nujiang catfish *Creteuchiloglanis macropterus* through a genome-wide single nucleotide polymorphisms resource generated by RAD-seq

**DOI:** 10.1038/s41598-018-27677-7

**Published:** 2018-06-21

**Authors:** Jingliang Kang, Xiuhui Ma, Shunping He

**Affiliations:** 1 The Key Laboratory of Aquatic Biodiversity and Conservation of Chinese Academy of Sciences, Institute of Hydrobiology, Chinese Academy of Sciences, Wuhan, Hubei 430072 China; 2 University of Chinese Academy of Sciences, Beijing, 100049 China; 3 College of Animal Science, Guizhou University, Guizhou, 550025 China

Correction to: *Scientific Reports* 10.1038/s41598-017-02853-3, published online 06 June 2017

In the original version of this Article, there were errors in Affiliations 1 and 2 which were incorrectly listed ‘The Key Laboratory of Aquatic Biodiversity and Conservation of Chinese Academy of Science, Institute of Hydrobiology, Chinese Academy of Science, Wuhan, Hubei, 430072, China’ and ‘Institute of Hydrobiology, University of Chinese Academy of Science, Beijing, 10001, China’ respectively. The correct affiliations are listed below;

Affiliation 1:

The Key Laboratory of Aquatic Biodiversity and Conservation of Chinese Academy of Sciences, Institute of Hydrobiology, Chinese Academy of Sciences, Wuhan, Hubei, 430072, China

Affiliation 2:

University of Chinese Academy of Sciences, Beijing, 100049, China

These errors have now been corrected in the PDF and HTML versions of the Article and in the accompanying Supplementary Information file.

