# Supplementary material for: Population genetics analysis of the Nujiang catfish Creteuchiloglanis macropterus through a genome-wide single nucleotide polymorphisms resource generated by RAD-seq
Source: Sci Rep. 2017 Jun 6;7:2813. doi: 10.1038/s41598-017-02853-3 (PMC5460224; doi:10.1038/s41598-017-02853-3)

Population genetics analysis of the Nujiang catfish *Creteuchiloglanis macropterus* through a genome-wide single nucleotide polymorphisms resource generated by RAD-seq

Jingliang Kang,[1,2] Xiuhui Ma,[3] and Shunping He[1]

[1]The Key Laboratory of Aquatic Biodiversity and Conservation of Chinese Academy of Sciences, Institute of Hydrobiology, Chinese Academy of Sciences, Wuhan, Hubei, 430072, China, [2]University of Chinese Academy of Sciences, Beijing, 100049, China, [3]College of Animal Science, Guizhou University, Guizhou, 550025, China. Correspondence and requests should be addressed to S.H. (email: clad@ihb.ac.cn)

Tables

Table S1. Genetic diversity in *C. macropterus* estimated from 2983bp mtDNA sequence (COI, Cytb, CR and ND5, considered sites with gaps).

	Mitochondrial DNA			
Pop. lables	N_{mt}	N_H	H	$\pi \pm SD$ (%)
QNT	15	2	0.419	0.00042 ±0.011
CW	12	8	0.848	0.00286 ±0.046
XYG	15	2	0.133	0.00004 ±0.004
LMD	15	6	0.743	0.00140 ±0.038
PH	18	9	0.850	0.00063 ±0.013
DLB	14	12	0.967	0.00151 ±0.044
LK	9	9	1	0.00162 ±0.035
ML	9	4	0.417	0.00120 ±0.062
Total	102	49	0.894	0.01251 ±0.066

Population labels and number of individuals (N_{mt}) analysed for mtDNA, the number of haplotypes (N_H), haplotype diversity (H) and nucleotide diversity ($\pi \pm SD$, in percent) were calculated.

Table S2. Pairwise F_{ST} using four mitochondria genes in 8 populations of *C. macropterus* in the Nujiang river.

Pop	CW	LMD	XYG	QNT	DLB	PH	LK	ML
CW	0							
LMD	**0.224**	0						
XYG	**0.271**	**0.270**	0					
QNT	**0.406**	**0.386**	**0.693**	0				
DLB	**0.916**	**0.941**	**0.968**	**0.961**	0			
PH	**0.941**	**0.961**	**0.985**	**0.978**	**0.160**	0		
LK	**0.903**	**0.944**	**0.986**	**0.974**	-0.005	**0.201**	0	
ML	**0.564**	**0.590**	**0.845**	**0.776**	**0.945**	**0.968**	**0.951**	0

Bold numbers are significant values under α=0.05.

Figures

Fig. S1. Median-joining network of concatenated mtDNA sequences (COI, Cytb, CR and ND5) haplotypes from eight populations of *Creteuchiloglanis macropterus*. The circle size of haplotype denotes the number of observed individuals. Colors correspond to different regions. White circles represent intermediate haplotypes not observed.

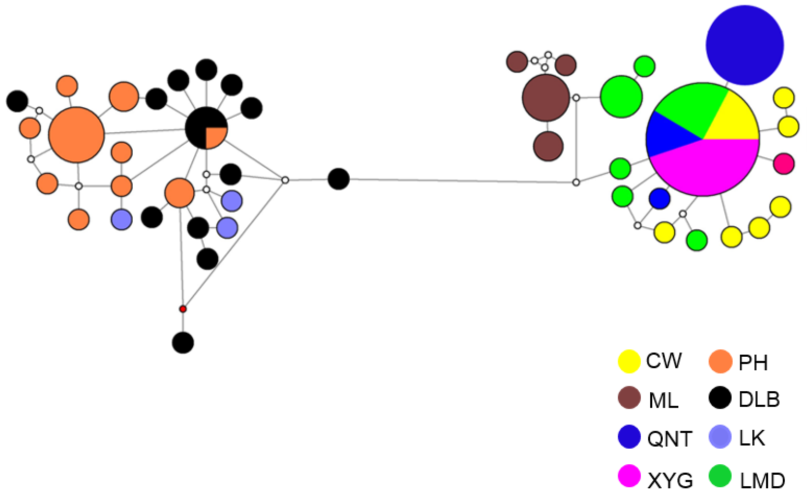

Fig. S2. Graph of ΔK = mean (| $L''(K)$ |) / sd ($L(K)$) as a function of K (potential number of genetic clusters).

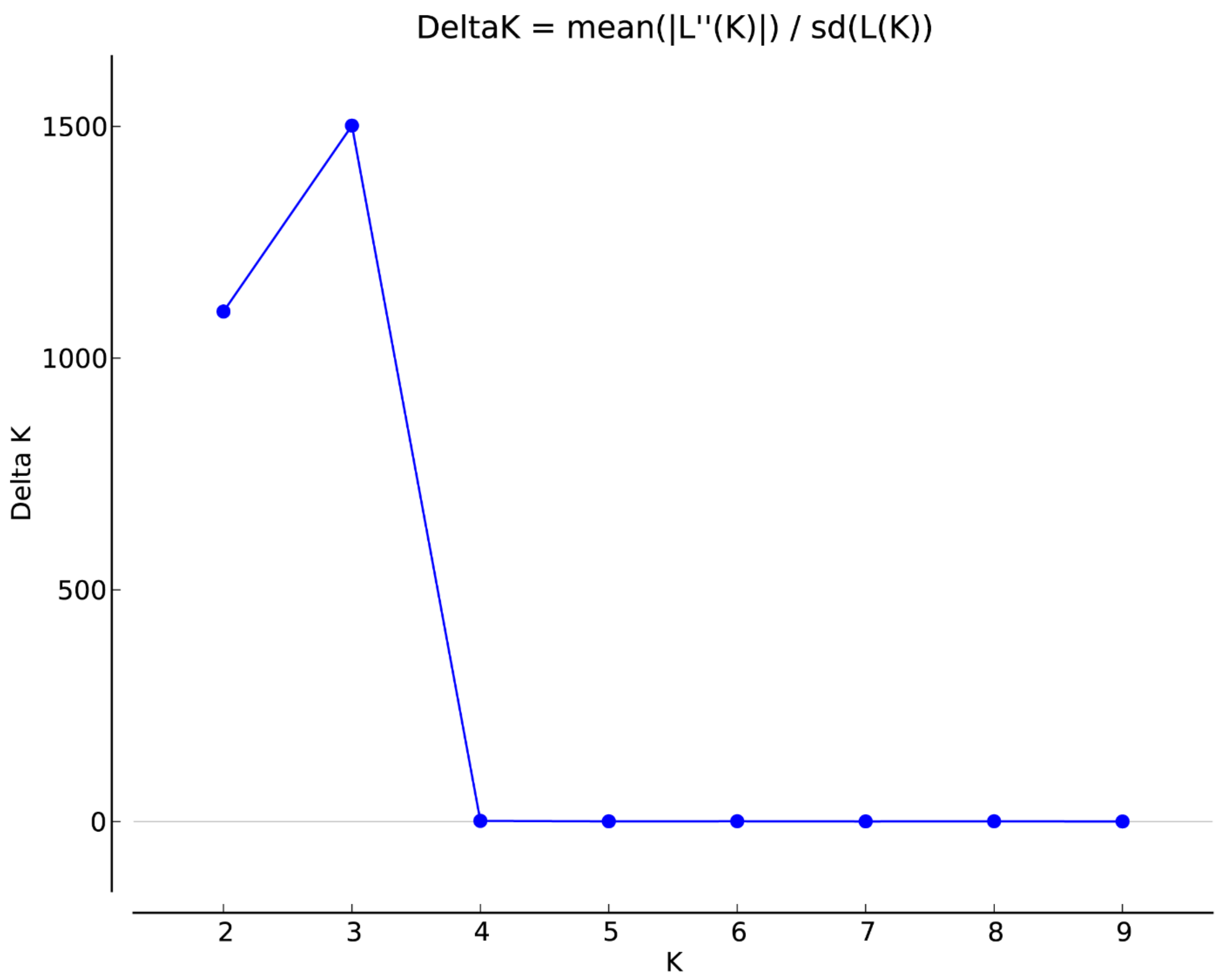

Fig. S3. Tajima's D values of 957 RAD tags in UT and LT populations, respectively. Red points stand for RAD tags of LT, blue points stand for RAD tags of UT.

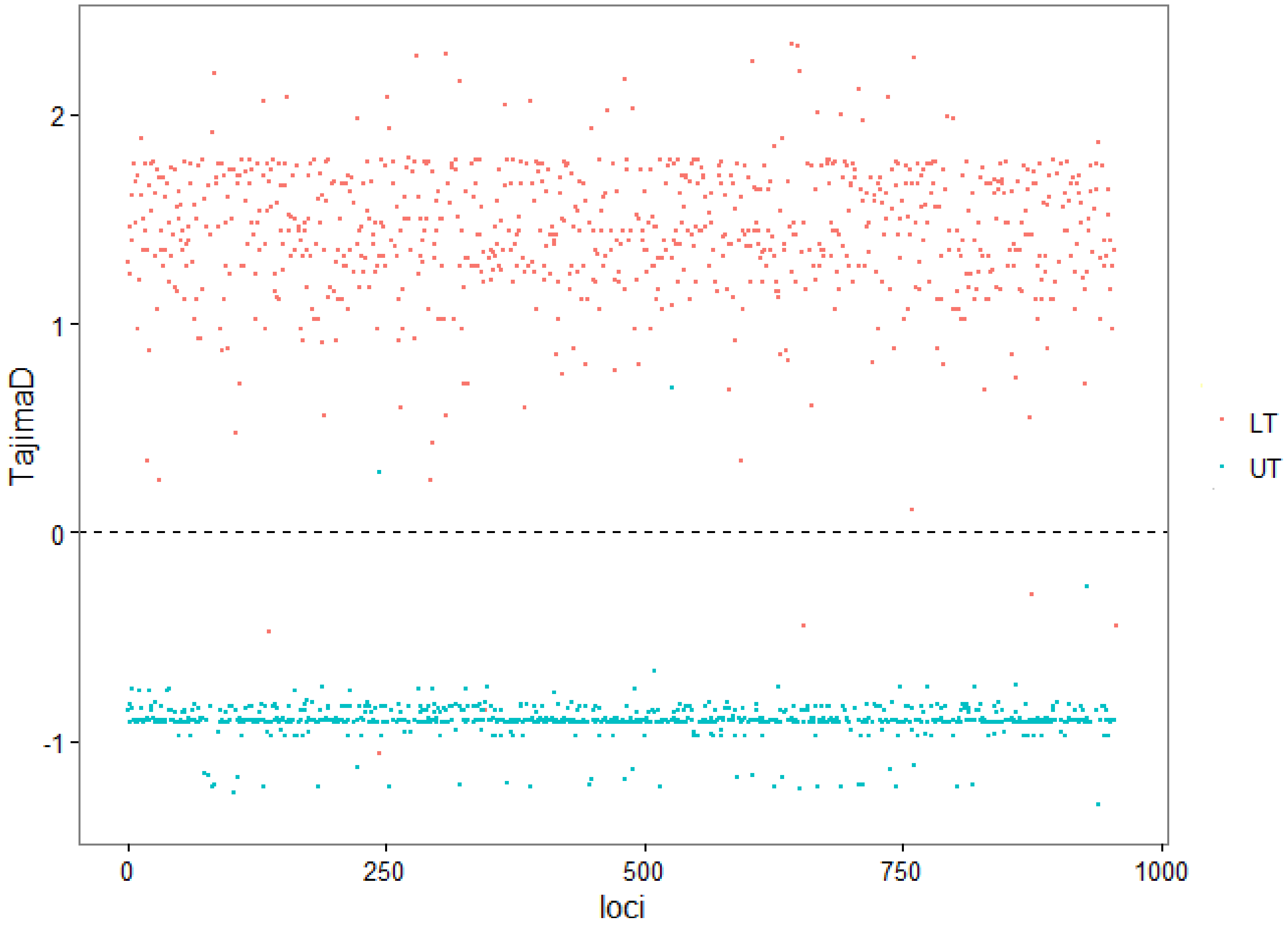

Fig. S4. Global outlier detection among 1,679 SNPs (dataset 1) in 10 *C. macropterus* populations from the Nujiang River. The vertical line represents a false discovery threshold of 0.01.

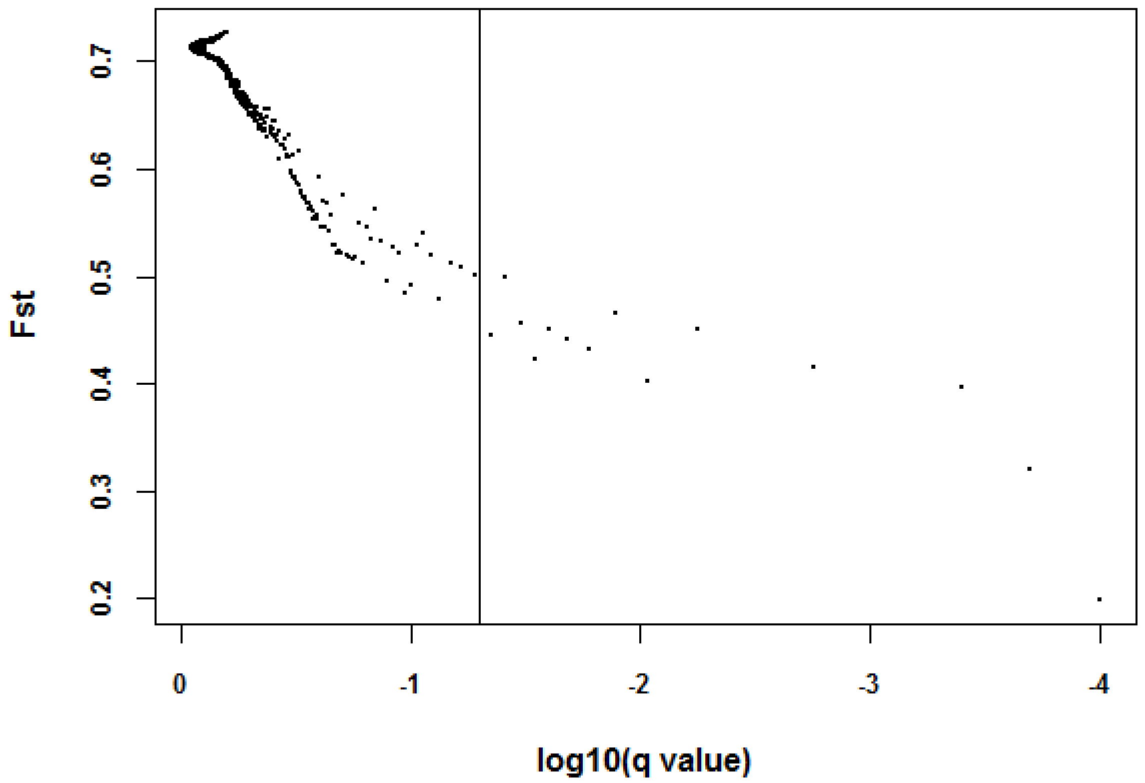

Fig.S5. Gene ontology assignment plot. The plot shows GO of candidate genes for adaptive differentiation (containing outlier SNPs) and genes with neutral SNPs in up-tributaries and low-tributary.

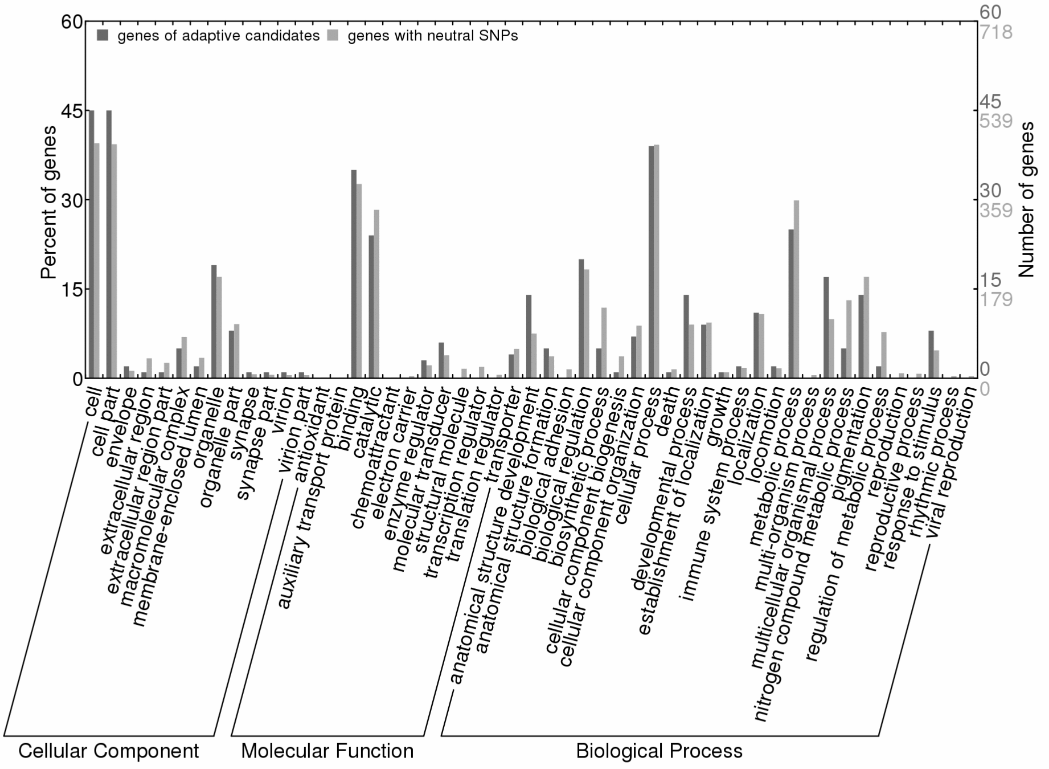

Fig.S6. Gene ontology assignment plot. The plot shows GO of candidate genes for adaptive differentiation (containing outlier SNPs) and genes with neutral SNPs in all tributaries and MS.

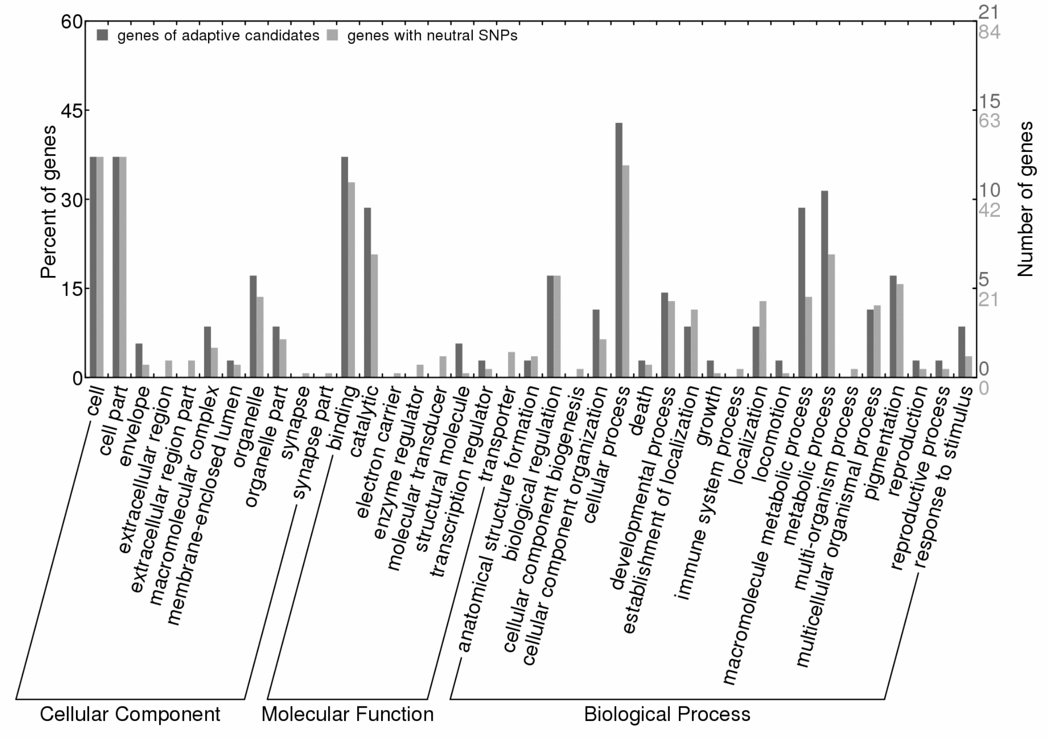

Fig.S7. Inference of five representative *C. macropterus* populations demographic history. DG and LK are two populations from MS, LMD is a mid-tributary, QNT and ML is a population of up-tributaries and low-tributaries, respectively.

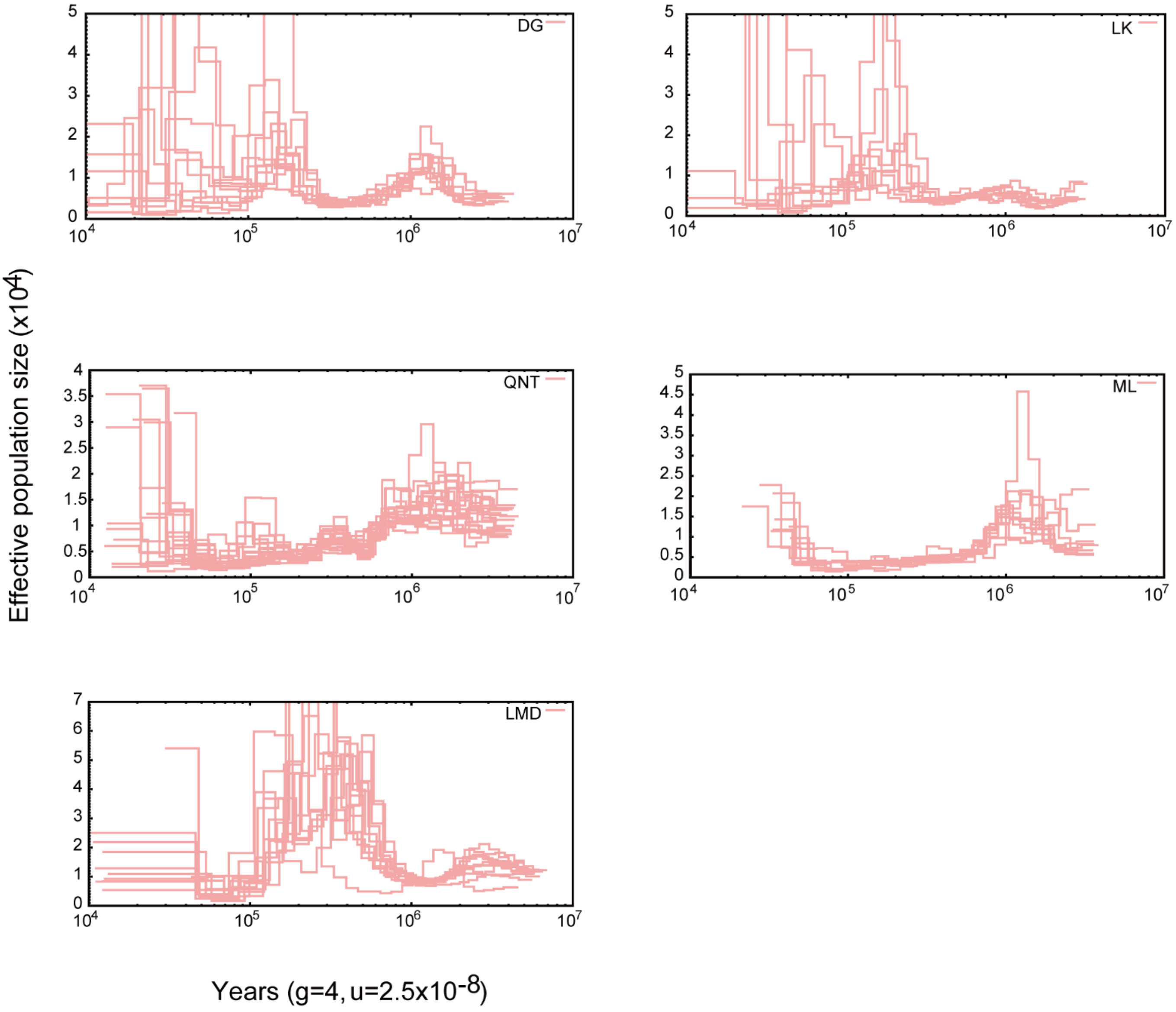

Fig. S8. Bayesian skyline plots (BSPs) estimated by BEAST for *C. macropterus*in MS (a), tributaries (b), and both MS and tributaries. The X-axis shows time in millions of years before present. Y-axis (logarithmic scale) indicates effective population size of females (N_e) estimates multiplied by generation time. The solid line indicates the median of population size, and the 95% HPD incredibility interval is depicted in blue.

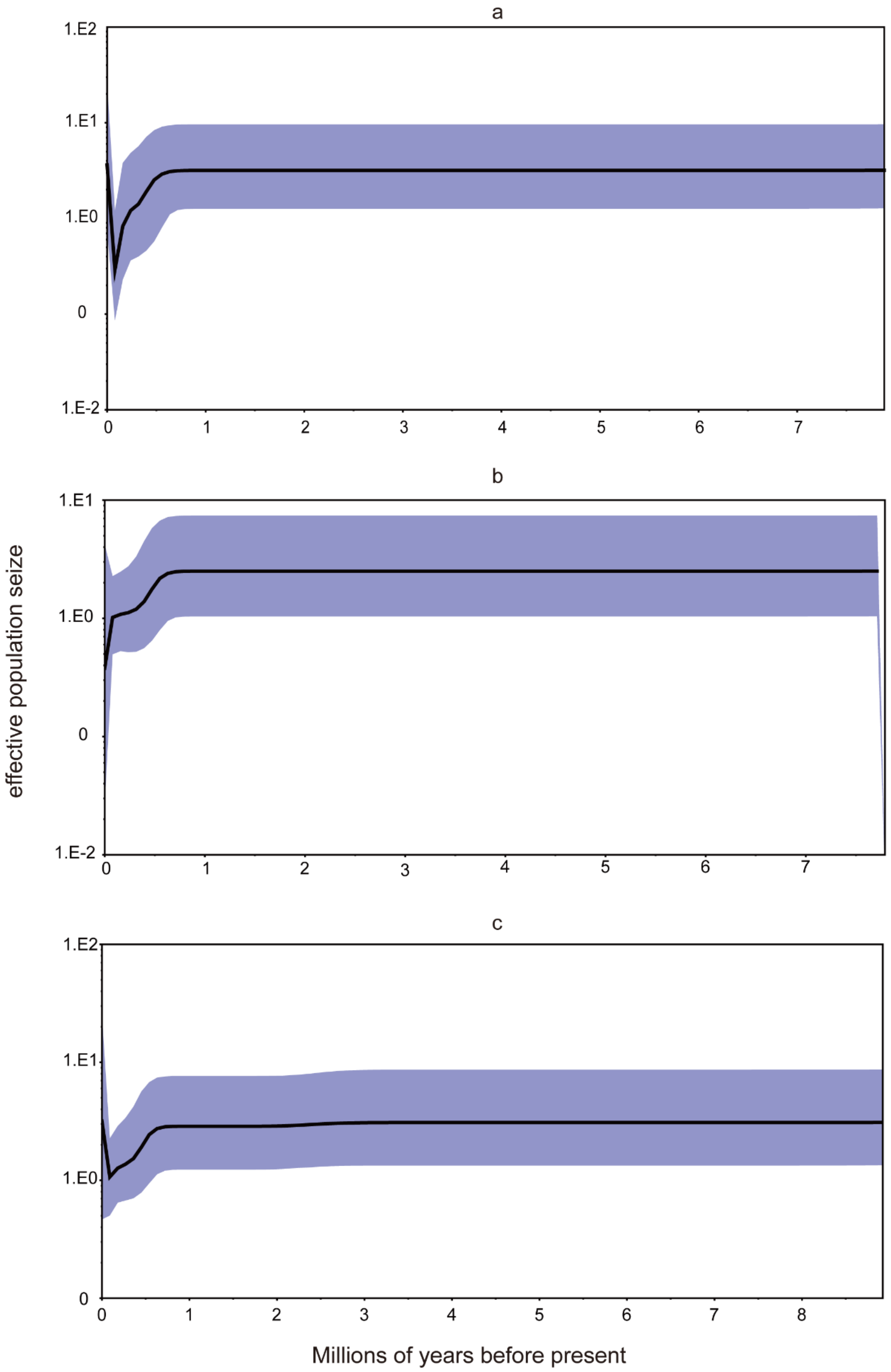

Supplement: Supplementary file 1 — supplementary information [file 41598_2017_2853_MOESM1_ESM.pdf]
